# The complete mitochondrial genome of the hybrid grouper *Epinephelus lanceolatus* (♀) × *E. akaara* (♂)

**DOI:** 10.1080/23802359.2018.1511842

**Published:** 2018-10-26

**Authors:** Jae Hoon Kim, Jong Yeon Park, Choong Hwan Noh, Min Joo Kang, Thai Quoc Truong, In-Chul Bang

**Affiliations:** a Department of Life Science & Biotechnology, Soonchunhyang University, Asan, Republic of Korea;; b Marine BioResources Research Center, Korea Institute of Ocean Science and Technology, Busan, Republic of Korea;; c Research Institute for Aquaculture No. 3, Mariculture Research and Development Center No. 2, Nha Trang, Vietnam

**Keywords:** *Epinephelus lanceolatus*; *E*. *akaara*, hybrid grouper, mitochondrial genome

## Abstract

This study determined the complete mitochondrial genome of the hybrid grouper *Epinephelus lanceolatus* ♀ × *E*. *akaara* ♂. The complete mitochondrial genome is 16,743 bp long and includes 13 protein-coding genes, two *ribosomal RN*A genes, 22 *transfer RNA* genes, and a control region. The nucleotide composition of the L-strand was as follows: A 20.00, C 14.10, G 29.21, and T 36.68%. All of the genes are encoded on the H-strand, except for NADH dehydrogenase subunit (ND6) and eight *tRNA* genes. A phylogenetic tree constructed using the maximum-likelihood (ML) method showed that the hybrid grouper *E. lanceolatus* ♀ × *E*. *akaara* ♂ is similar to *E*. *lanceolatus*.

There are more than 100 species of grouper worldwide, with most inhabiting Asian seas. Both *Epinephelus akaara* and *Epinephelus* *lanceolatus* belong to Serranidae in the order Perciformes. Hybrid groupers have faster growth rates, greater disease resistance, and higher economic value than purebred groupers. Many grouper hybrids are currently being studied (Chen et al. 2017; Tang et al. 2016). This study determined basic data on the Serranidae. We report the complete mitogenome of the novel hybrid grouper *E*. *lanceolatus* (♀) × *E*. *akaara* (♂), which was developed in Tongyeong, Republic of Korea (34°49′36.8′′N 128°20′01.6′′E). Fry of the novel hybrid grouper was preserved in 70% ethyl alcohol immediately after hatching in October 2017.

The complete mitochondrial genome of the hybrid grouper has 16,743 bp and consisted of 13 protein-coding genes, 22 *transfer RNA* genes, two *ribosomal RNA* genes, and the control region (D-loop). The encoded genes are similar to those of other Serranidae (Kim et al. 2016; Tang et al. 2016, 2017; Chen et al. 2017). The total length of the 13 protein-coding genes is 11,429 bp or 68.26% of the mitochondrial genome. The nucleotide composition of the L-strand is as follows: A 20.00, C 14.10, G 29.21, and T 36.68%. The G + C content (43.31%) is lower than the A + T content (56.68%). All of the genes are encoded on the H-strand, except for NADH dehydrogenase subunit (ND6) and eight tRNA genes. Most of the coding sequences start with ATG, but COXI and ATP6 start with GTG and CTG, respectively. The 22 transfer RNA genes range from 68 to 76 bp in length. Eight of the protein-coding genes (ND1, COXI, ATP8, ATP6, COXIII, ND4L, ND5, and ND6) stop with TAA, while ND2, COXII, ND3, ND4, and CYTB end with an incomplete T. The 12S ribosomal RNA is between transfer RNA-Phe and transfer RNA-Val and is 953 bp in length. The 16S ribosomal RNA is between transfer RNA-Val and transfer RNA-Leu and is 1706 bp in length. The control region (D-loop) is 1041 bp long and located between tRNA-Pro (TGG) and tRNA-Phe (GAA). A phylogenetic tree was constructed based on the complete mitochondrial genome and that of other hybrid groupers using the maximum-likelihood (ML) method. Five species of *Cephalopholis* and *Hyporthodus* were used as an outgroup. The phylogenetic tree showed that hybrid *E*. *lanceolatus* ♀ and *E*. *akaara* ♂ have a close relationship with *E*. *lanceolatus*. This study will be an important source of data for *Epinephelus* species (Figure 1).

**Figure 1.**
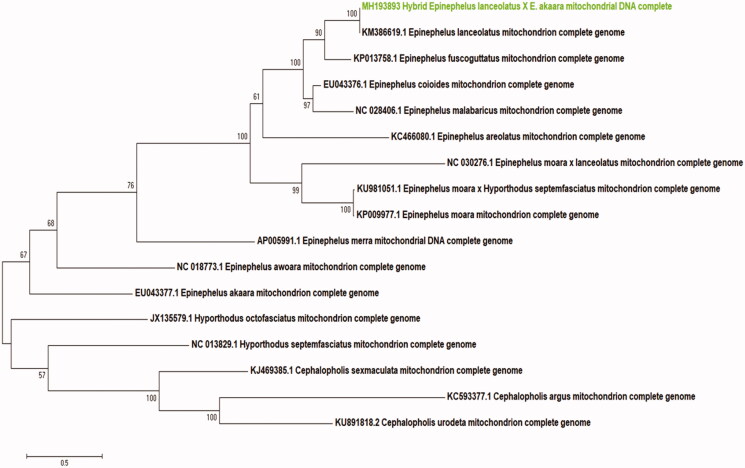
Phylogenetic tree of hybrid grouper *E. lanceolatus* (♀) × *E. akaara* (♂) with 11 *Epinephelus* and 5 out group. The number of each nod is the bootstrap probability. Phylogenetic tree was constructed by Maximum-likelihood (Kumar et al. 2004).
